# Role of HMGB1 in ischemia/reperfusion injury in a rat model of myocardial infarction

**DOI:** 10.1038/s41598-025-28160-w

**Published:** 2025-11-25

**Authors:** Martina Cebova, Andrej Barta, Katarina Bujnova, Olga Pechanova

**Affiliations:** 1 Department of Neuro-Cardiovascular Interaction, Institute of Normal and Pathological Physiology, Centre of Experimental Medicine, Slovak Academy of Sciences, Sienkiewiczova 1, 813 71 Bratislava, Slovakia; 2 Institute of Pathophysiology, Faculty of Medicine, Comenius University, 811 08 Bratislava, Slovakia

**Keywords:** Myocardial infarction, High mobility group box 1, Nitric oxide, NFκB, TLR4, Biochemistry, Cardiology, Medical research

## Abstract

Myocardial infarction (MI) results from the abrupt interruption of coronary blood flow, leading to ischaemia and cardiac tissue necrosis. High mobility group box 1 (HMGB1), a damage-associated molecular pattern, plays a dual role in MI, offering protection or causing damage depending on the cellular redox status, timing of its activity, and location. The aim of this study was to analyse the effects of an anti-HMGB1 protein on certain biochemical parameters of individual myocardial zones, the infarct zone (IZ), injured zone (INZ), and noninfarct zone (NIZ), after MI. Twelve-week-old male WKY rats were randomly divided into 3 experimental groups: 1. control (sham), 2. MI (MI), and 3. MI plus anti-HMGB1 treatment (MI + aHMGB1). MI was induced via 20-min ligation of the left descending coronary artery and was followed by reperfusion. Anti-HMGB1 (10 μl) was administered before reperfusion. The animals were sacrificed 7 days later. The plasma levels of the cytokines IL-6 and TNF-α were determined with a Bio-Plex Pro Cytokine Kit. Total NO-synthase activity was determined by the conversion of [^3^H]-arginine to [^3^H]-citrulline. Protein expression was analysed via Western blotting. Lipid peroxidation was evaluated by determining conjugated diene concentrations in the heart, while collagen content in the left ventricle was quantified by measuring hydroxyproline. Anti-HMGB1 treatment increased both NOS activity and endothelial NOS expression while reducing inducible NOS and NFκB expression. It also decreased plasma IL-6 and TNF-α levels, reduced lipid peroxidation, and lowered collagen content. The anti-HMGB1 protein demonstrated cardioprotection by modulating inflammation and oxidative stress, suggesting its potential as a therapeutic agent for MI and related cardiovascular conditions.

**Supplementary Information:**

The online version contains supplementary material available at 10.1038/s41598-025-28160-w.

## Introduction

Myocardial infarction (MI) is a severe cardiovascular event caused by the sudden blockage of a coronary artery, leading to ischaemia and myocardial tissue necrosis. Despite therapeutic advances, MI remains a major cause of morbidity and mortality, contributing significantly to the 32% of deaths worldwide attributed to cardiovascular diseases^1^. The primary aetiology of MI is atherosclerosis, a chronic inflammatory disease characterized by lipid accumulation and plaque formation in the arterial wall. Risk factors such as dyslipidaemia, smoking, hypertension, and hyperglycaemia contribute to endothelial dysfunction, which is central to the pathogenesis of atherosclerosis^2^. Ischaemia induces necrosis, apoptosis, and autophagy in cardiomyocytes, which lead to fibrotic tissue deposition, impaired cardiac contractility, and increased susceptibility to heart failure.

Oxidative stress is a major contributor to the above pathological process. The ischaemic myocardium experiences a surge in reactive oxygen species (ROS), especially upon reperfusion. Paradoxically, reperfusion exacerbates myocardial damage through increased ROS generation, resulting in lipid peroxidation, protein oxidation, and DNA damage, all of which compromise myocardial function and contribute to adverse ventricular remodelling.

MI also triggers a robust inflammatory response, which is necessary for tissue repair; however, excessive or sustained immune activation can amplify myocardial injury^3^. Damaged or necrotic cardiomyocytes release danger-associated molecular patterns (DAMPs), which activate immune signalling pathways, among which high-mobility group box protein 1 (HMGB1) has emerged as a key mediator in the post-MI inflammatory cascade^4^. The HMGB1 protein is normally found in the nucleus and is involved in chromatin organization and DNA repair, but when released extracellularly, HMGB1 acts as a potent proinflammatory signal. HMGB1 interacts with pattern recognition receptors such as Toll-like receptor 4 (TLR4) and the receptor for advanced glycation end products (RAGE), activating the nuclear factor kappa B (NFκB) pathway. NFκB translocation into the nucleus induces the expression of proinflammatory cytokines, including tumor necrosis factor-alpha (TNF-α) and interleukin-6 (IL-6), as well as various chemokines^5,6^. Elevated serum levels of HMGB1 in MI patients correlate with increased risks of mortality and impaired ventricular function and reduced exercise capacity^7^. The role of HMGB1 in MI remains complex and context dependent, and experimental models have produced conflicting results. In models of permanent coronary artery ligation, HMGB1 appears to exert cardioprotective effects, enhancing angiogenesis and limiting fibrosis. For example, Takahashi et al.^8^ reported improved left ventricular function and reduced myocardial fibrosis following HMGB1 administration. In contrast, studies using ischaemia‒reperfusion models suggest that HMGB1 plays a detrimental role. Kohno et al.^9^ demonstrated that HMGB1 inhibition reduced inflammation but led to infarct scar thinning and adverse remodelling. Similarly, Andrassy et al.^10^ reported that HMGB1 antagonism improved cardiac function postinjury, whereas recombinant HMGB1 administration impaired cardiac function. These opposing outcomes may reflect the different injury mechanisms and the timing of HMGB1 activity. In permanent ischaemia, the angiogenic properties of HMGB1 may be beneficial.

The aim of this study was to evaluate the effects of anti-HMGB1 protein administration on biochemical and morphological parameters in 12-week-old male WKY rats following MI. We specifically analysed nitric oxide (NO) levels, oxidative stress markers, and the expression of proteins related to HMGB-1 and antioxidant defence in distinct cardiac zones. We hypothesized that anti-HMGB1 treatment would ameliorate the oxidative and inflammatory changes associated with myocardial infarction, potentially offering a therapeutic strategy to mitigate cardiac damage.

## Materials and methods

### Animals and treatment

The experiments were carried out with male 12-week-old Wistar Kyoto rats (270–300 g), housed under constant temperature (22 ± 2 °C) and relative humidity (55 ± 10%) conditions on a 12-h light/dark cycle. The animals were obtained from Velaz Laboratories (Czech Republic). All procedures and experimental protocols were performed in accordance with the institutional guidelines and were approved by the State Veterinary and Food Administration of the Slovak Republic (Ro-4430/13-221 and Ro-4279/16-221) and by the ethical committee of the Centre of Experimental Medicine SAS according to the European Convention for the Protection of Vertebrate Animals used for Experimental and other Scientific Purposes, Directive 2010/63/EU of the European Parliament. The study was conducted and the results are reported in accordance with the ARRIVE guidelines (https://arriveguidelines.org).

The following three experimental groups were randomly established: (1) Control sham-operated 12-week-old male WKY rats (sham), (2) Age-matched male WKY rats with experimentally induced myocardial infarction (MI), and (3) Age-matched male WKY rats with experimentally induced myocardial infarction administered anti-HMGB1 protein at a dose of 10 µl (MI + a-HMGB1) (n = 8 in each group). The anti-HMGB1 protein (1 mg/mL, 10 µL per rat; approximately 1 mg/kg) was administered locally by direct intramyocardial injection at the onset of reperfusion to achieve targeted neutralization of HMGB1 signalling with minimal systemic exposure. The selected dose was based on previously published studies demonstrating effective inhibition of HMGB1-mediated inflammation in rodent models of ischemia/reperfusion at similar doses^11,12^. Preliminary experiments with a double dose (20 µL; ~ 2 mg/kg) resulted in 100% mortality, indicating that 10 µL represents the maximal safe and biologically active concentration. All the animals had free access to drinking water and standard laboratory chow (Altromin 1324P). The blood pressure of each prewarmed animal was measured via noninvasive tail-cuff plethysmography using an MRBP blood pressure system (The IITC Life Science) before surgery and 7 days after experimentally induced myocardial infarction.

### Experimentally induced myocardial infarction

**Fig. 1:**
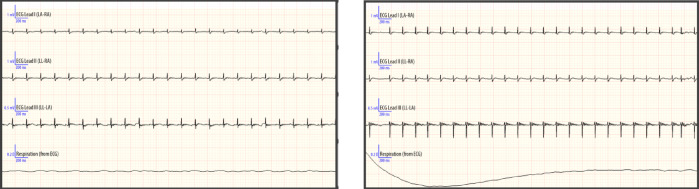
Representative ECGs from sham-operated animals (left) and animals with induced myocardial infarction (right).

Experimental MI was induced in 12-week-old normotensive WKY rats by left descending coronary artery ligation as previously described by Kosutova et al.^13^. Briefly, prior to surgery, 2 mg/kg butorphanol (s.c.) + 2 mg/kg meloxicam and 5 ml saline + 5% glucose (s.c.) were administered for analgesia. After the induction of anaesthesia (tiletamine–zolazepam; Zoletil 50, Virbac, France; 30 mg/kg i.p.), the rats were intubated and ventilated with a pressure-controlled rodent respirator at 70 strokes per minute. During the entire procedure, the rats were placed on a heating pad to maintain their body temperature at 37 °C. Left lateral thoracotomy was performed through the fifth intercostal space. After the pericardium was opened, the left coronary artery was reversibly ligated with 5–0 silk, and C-2 suture thread (Ethicon, San Lorenzo, USA) was applied approximately 2 mm from its origin. MI was confirmed by ECG. Twenty minutes after MI induction but prior to reperfusion, 10 μl of the anti-HMGB1 protein solution was administered directly into the myocardium. Thereafter, the thoracotomy was closed in layers using Vicryl 4-0; SH-1 and Prolene 8-0; and CC (Ethicon, San Lorenzo, USA). Sham-operated animals were subjected to the same procedure except for ligation. MI was confirmed using electrocardiography (ECG). ECGs were obtained from 4 needle electrodes that had been subcutaneously inserted into the standard left–right axillar and groin sites with the rat in the supine position (Rodent Surgical Monitor+, Indus Instruments, Webster, Texas, USA). Representative ECGs from the sham-operated and myocardial infarction groups are presented in Fig. 1. Seven days after MI induction, the animals were sacrificed by an overdose of anaesthesia, and their BW (body weight), HW (heart weight) and kidney weight (KW) were determined. The plasma concentrations of TNF-α and IL-6 were quantified using a commercially available Bio-Plex Pro™ Rat Cytokine Assay Kit (Bio-Rad, Hercules, CA, USA). Cardiac troponin I levels were measured with a Rat Cardiac Troponin I SimpleStep ELISA Kit (Abcam, Cambridge, UK).

### Total NOS activity

Total NOS activity was determined in 20% crude tissue homogenates from different parts of the heart, namely, the infarct zone (IZ), injured zone (INZ) and noninfarct zone (NIZ), by measuring the formation of [3H]L-citrulline from [3H]L-arginine (ARC, St. Louis, MT, USA) as previously described by Cebova et al.^14^. The [3H]L-citrulline content was measured by liquid scintillation counting using a Quanta Smart TriCarb Liquid Scintillation Analyser (Perkin-Elmer, Waltham, MA, USA). NOS activity is expressed as picokatal per gram of protein (pkat/g protein).

### Protein expression of eNOS, iNOS, NFκB, and TLR4

Tissue samples from each myocardial zone investigated were homogenized in lysis buffer (0.05 mM Tris containing protease inhibitor cocktail; Sigma‒Aldrich, Germany). After centrifugation (15,000 rpm at 4 °C for 20 min), the protein concentrations were determined via the Lowry assay. The supernatants were subjected to SDS‒PAGE on 12% gels to examine the proteins, which were subsequently transferred to nitrocellulose membranes. The membranes were blocked with 5% nonfat milk in Tris buffer solution (TBS; pH 7.6) containing 0.1% Tween-20 (TBS-T) for 1 h at room temperature and probed with primary polyclonal rabbit anti-endothelial NOS and anti-inducible NOS, anti-NFκB and anti-TLR4 antibodies and an anti-GAPDH antibody as a loading control (Abcam, Cambridge, UK) overnight at 4 °C. Antibodies were detected by incubation with a secondary peroxidase-conjugated anti-rabbit antibody (Abcam, Cambridge, UK) at room temperature for 2 h. The intensities of the bands were visualized using an enhanced chemiluminescence system (Bio-Rad, Hercules, CA, USA), quantified using a ChemiDocTM Touch Imaging System (Image LabTM Touch software, version 5.2; Bio-Rad, Hercules, CA, USA) and normalized to the intensities of the GAPDH bands in each individual part of the heart.

### Measurement of the conjugated diene content

The concentration of conjugated dienes was assessed in lipid extracts obtained from the left ventricle. The tissue samples were homogenized in 15 mmol/L EDTA containing 4% NaCl. Lipid extraction was performed using a 1:1 (v/v) chloroform–methanol solution. Following extraction, the chloroform was evaporated under a nitrogen atmosphere, and the residue was redissolved in cyclohexane (PanReac AppliChem, Darmstadt, Germany). Conjugated diene levels were measured spectrophotometrically at 233 nm using a NanoDrop One spectrophotometer (Thermo Fisher Scientific, Waltham, MA, USA). The concentrations were calculated using an extinction coefficient of ε = 29,000 L·mol⁻^1^·cm⁻^1^ and are expressed as nanomoles per gram of tissue.

### Measurement of collagen content

Collagen content within the myocardial tissue was indirectly assessed through the quantification of hydroxyproline (Hyp), as a reliable biochemical marker of total collagen levels. A modified protocol based on the method originally described by Reddy and Enwemeka^15^ was used. For the analysis, homogenates from the left ventricle were used. Briefly, 25 µL aliquots of tissue homogenate were subjected to alkaline hydrolysis by the addition of 50 µL of 2 M NaOH. The mixture was then incubated at 110 °C for 60 min, promoting hydrolytic degradation of tissue proteins and liberating free hydroxyproline residues from collagen fibers. Post-hydrolysis, samples were cooled and subsequently neutralized, followed by oxidation using 450 µL of chloramine-T reagent at ambient temperature for 25 min. To facilitate chromogenic detection, 500 µL of p-dimethylaminobenzaldehyde (DMAB) solution was added, and the reaction mixture was incubated at 65 °C for 20 min. The resulting chromophore was quantified spectrophotometrically at 550 nm using a NanoDrop One instrument (Thermo Fisher Scientific, Waltham, MA, USA). Collagen concentrations were extrapolated from a hydroxyproline standard curve and normalized to total protein content in each homogenate sample. Measurements were conducted in triplicate to ensure reproducibility. A conversion factor was applied, assuming hydroxyproline comprises approximately 12.5% of the collagen molecule by mass.

### Statistical analysis

The data are expressed as the mean ± S.E.M. values. One-way ANOVA and the Bonferroni post-hoc correction were used for statistical analysis. Differences between means were considered statistically significant at *p* < 0.05. The data were analysed using STATISTICA 10.

## Results

### Somatic parameters, blood pressure, and cytokine and troponin levels

Body weight, heart weight and kidney weight did not differ between the groups after coronary ligation. Additionally, there were no differences in other parameters, such as tibia length and the heart weight-to-body weight ratio (Table 1).

**Table 1:** Somatic parameters of sham-operated WKY rats (Sham), WKY rats with experimentally induced myocardial infarction (MI), and WKY rats with experimentally induced myocardial infarction administered anti-HMGB1 (MI + aHMGB1).

Parameter	Sham	MI	MI + aHMGB1
BW (g)	282.90 ± 5.55	283.67 ± 7.46	270.00 ± 4.01
HW (g)	1.04 ± 0.03	1.01 ± 0.03	0.95 ± 0.03
KW (g)	0.95 ± 0.04	0.94 ± 0.02	0.93 ± 0.05
TL (mm)	41.33 ± 1.38	40.50 ± 1.50	41.17 ± 0.91
HW/BW (g) × 10^−3^	3.67 ± 0.01	3.56 ± 0.02	3.52 ± 0.02

Body weight (BW), heart weight (HW), kidney weight (KW), tibia length (TL), and ratio of heart weight to body weight (HW/BW); Statistical significance as revealed by one-way ANOVA with subsequent Bonferroni post-hoc test when appropriate. n = 8. The data are the means ± SEMs.

No significant differences were found in the BP values among the control and both experimental groups (with or without aHMGB-1) after MI. We evaluated plasma cytokine levels before and after MI. As shown in Table 2, the levels of tumor necrosis factor alpha (TNF-α) and interleukin 6 (IL-6) were greater in the MI group than in the control group (*p* < 0.01). The administration of aHMGB-1 decreased TNF-α and IL-6 levels compared with those in the control group. (Table 2). The troponin level after myocardial infarction was significantly greater than that in the control group, as expected. On the other hand, aHMGB-1 administration did not affect the troponin level (Table 2).

**Table 2:** Blood pressure and cytokine and troponin levels of sham-operated WKY rats (Sham), WKY rats with experimentally induced myocardial infarction (MI), and WKY rats with experimentally induced myocardial infarction and anti-HMGB1 administration (Mi + aHMGB1).

Parameter	Sham	MI	MI + aHMGB1
BP (mmHg)	122 ± 1.8	12.1 ± 3.9	111 ± 2.5
TNF-α (pg/ml)	13.32 ± 2.1	28.2 ± 1.4*	14.9 ± 1.3#
IL-6 (pg/ml)	24.2 ± 5.2	46.4 ± 3.1*	28.1 ± 2.1#
cTnT (ng/ml)	6.23 ± 0.13	39.82 ± 0.21*	38.75 ± 0.22*

Blood pressure (BP), tumor necrosis factor alpha (TNF-α), interleukin 6 (IL-6), and troponin (cTnT); Statistical significance as revealed by one-way ANOVA with subsequent Bonferroni post-hoc test when appropriate: *p < 0.01 versus Sham; #*p* < 0.01 versus MI; n = 8. The data are the means ± SEMs.

### Total NOS activity

**Fig. 2:**
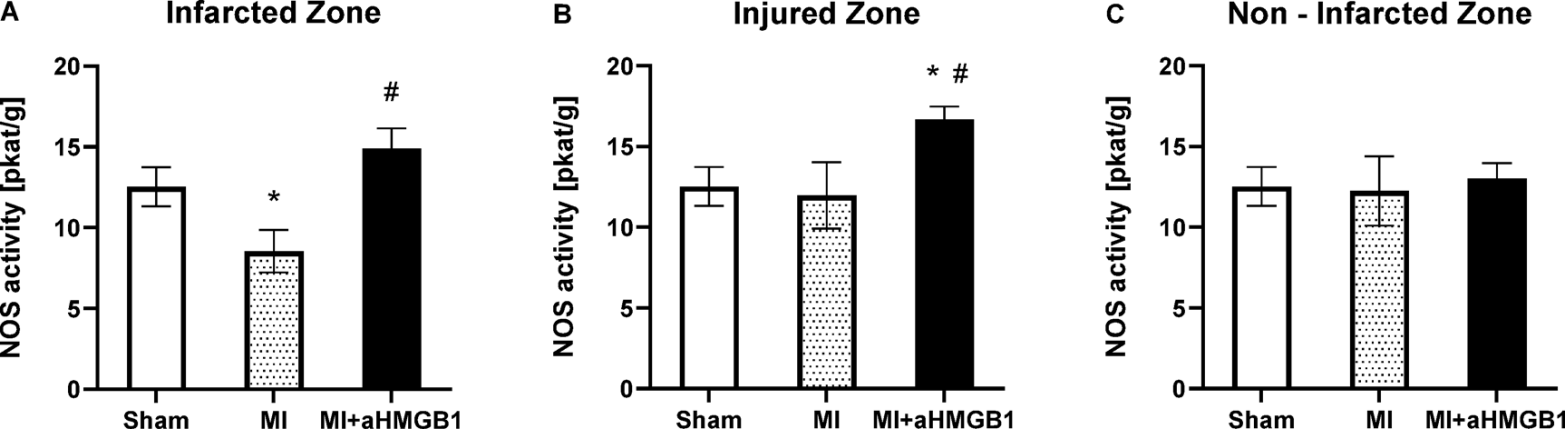
Total nitric oxide synthase (NOS) activity in the infarct zone (IZ; **A**), injured zone (INZ; **B**) and nonischaemic zone (NIZ; **C**) of the myocardium of sham-operated WKY rats (Sham), WKY rats with experimentally induced myocardial infarction (MI), and WKY rats with experimentally induced myocardial infarction administered anti-HMGB1 (MI + aHMGB1). Statistical significance as revealed by one-way ANOVA with subsequent Bonferroni post-hoc test when appropriate: *p < 0.01 versus Sham; #*p* < 0.01 versus MI; n = 8. The data are the means ± SEMs.

In the IZ, total NOS activity was significantly lower in the experimental myocardial infarction group than in the sham-operated control group (*p* < 0.01) (Fig. 2A). No significant differences in NOS activity were observed in the INZ or NIZ between the MI and sham groups. Interestingly, compared with sham animals, the administration of the anti-HMGB1 protein (MI + aHMGB1 group) led to a significant increase in the total NOS activity in the INZ (*p* < 0.01). Moreover, in both the IZ and INZ, total NOS activity was significantly elevated in the MI + aHMGB1 group compared with that in the MI group (*p* < 0.01), suggesting modulation via HMGB1 blockade. In the NIZ, no significant differences were observed among the experimental groups (Fig. 2A–C).

### Protein expression

**Fig. 3:**
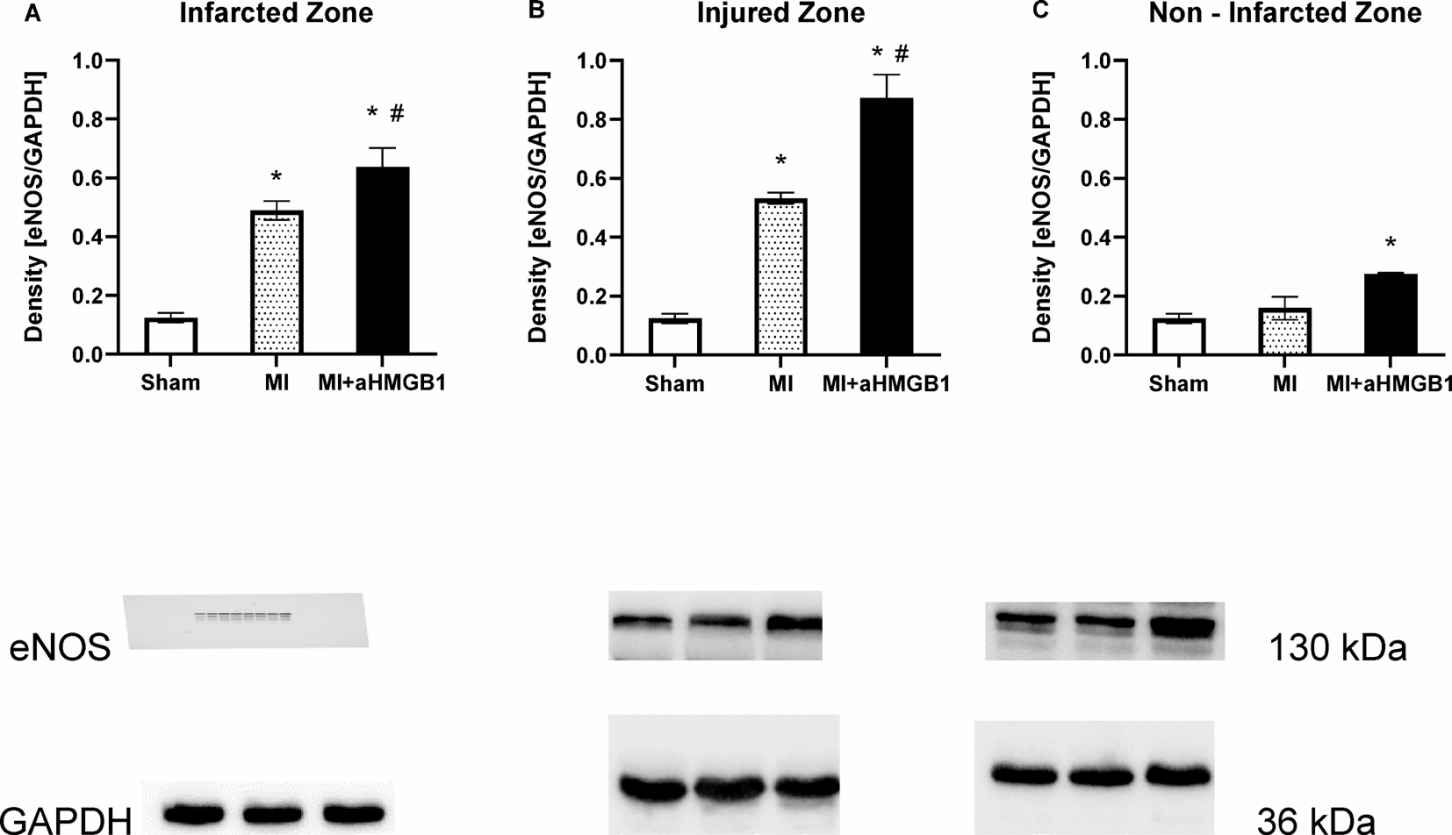
Expression of endothelial nitric oxide synthase (eNOS) in the infarct zone (IZ; **A**), injured zone (INZ; **B**) and nonischaemic zone (NIZ; **C**) of the myocardium of sham-operated WKY rats (Sham), WKY rats with experimentally induced myocardial infarction (MI), and WKY rats with experimentally induced myocardial infarction administered anti-HMGB1 (MI + aHMGB1). Statistical significance as revealed by one-way ANOVA with subsequent Bonferroni post-hoc test when appropriate: *p < 0.01 versus Sham; #*p* < 0.01; versus MI; n = 8. The data are the means ± SEMs.

Western blot analysis revealed changes in the protein expression of endothelial NOS (eNOS) in different zones of cardiac tissue. ENOS expression was significantly greater in both the IZ and INZ of MI animals than in those of sham-operated controls (*p* < 0.01) (Fig. 3). No significant differences in protein expression levels were detected in the NIZ. Compared with the MI (*p* < 0.01) and sham groups (*p* < 0.01), treatment with anti-HMGB1 further increased eNOS expression in the IZ and INZ, with the increase in the INZ being particularly pronounced (*p* < 0.01). In the NIZ, eNOS expression was also significantly elevated in the MI + aHMGB1 group compared with both the sham and MI groups (*p* < 0.01) (Fig. 3A–C).

**Fig. 4:**
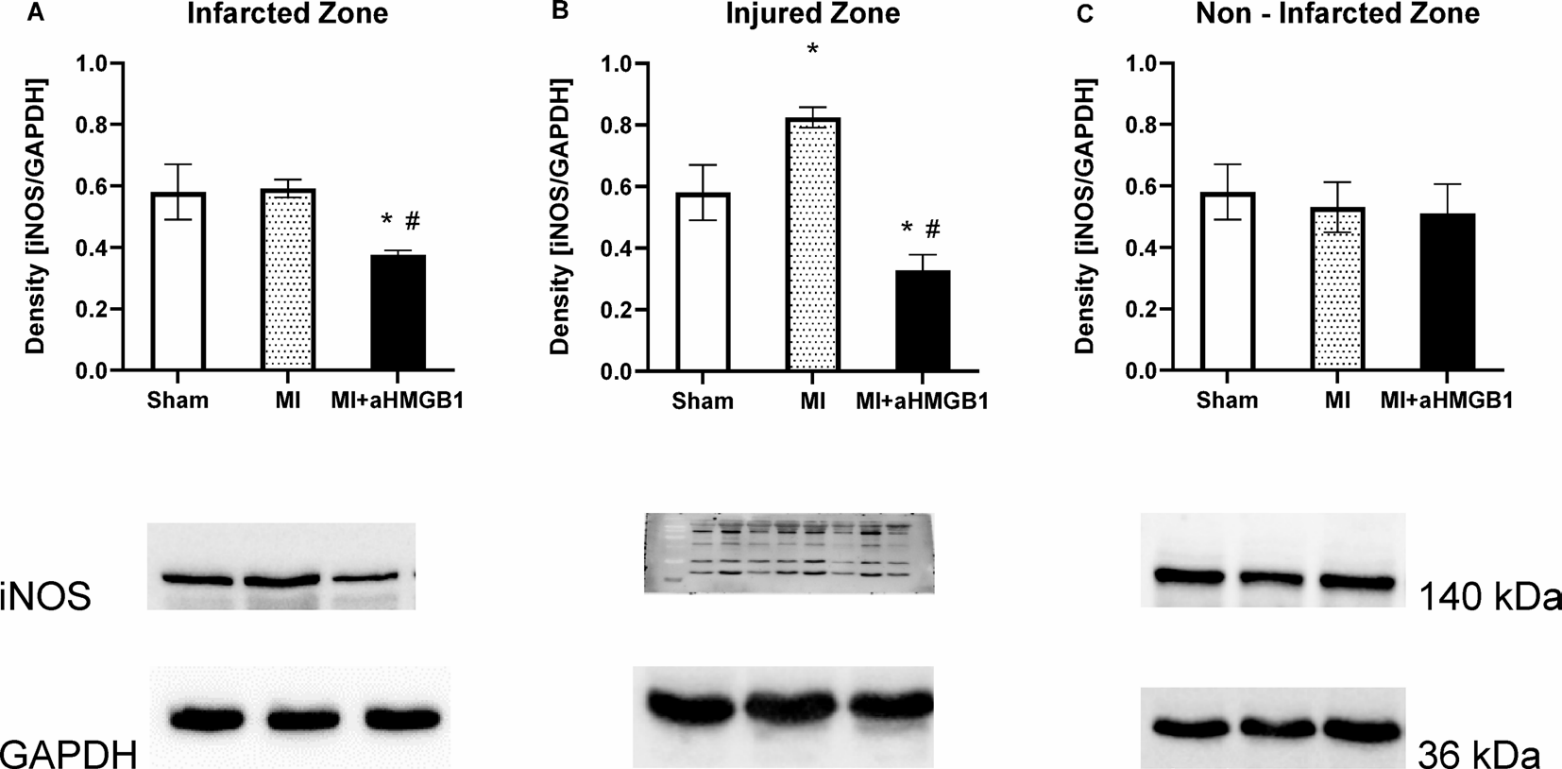
Expression of inducible nitric oxide synthase (iNOS) in the infarct zone (IZ; **A**), injured zone (INZ; **B**) and nonischaemic zone (NIZ; **C**) of the myocardium of sham-operated WKY rats (Sham), WKY rats with experimentally induced myocardial infarction (MI), and WKY rats with experimentally induced myocardial infarction administered anti-HMGB1 (MI + aHMGB1). Statistical significance as revealed by one-way ANOVA with subsequent Bonferroni post-hoc test when appropriate: *p < 0.01; versus Sham; #*p* < 0.01 versus MI; n = 8. The data are the means ± SEMs.

The expression of inducible NOS (iNOS) was markedly increased only in the INZ zone in the MI group compared with that in the sham control group (*p* < 0.01) (Fig. 4A–C). Anti-HMGB1 treatment significantly reduced iNOS levels in the IZ and INZ zones compared with those in both the control and MI groups (*p* < 0.01). In the NIZ of the myocardium, no changes in iNOS expression were detected.

**Fig. 5:**
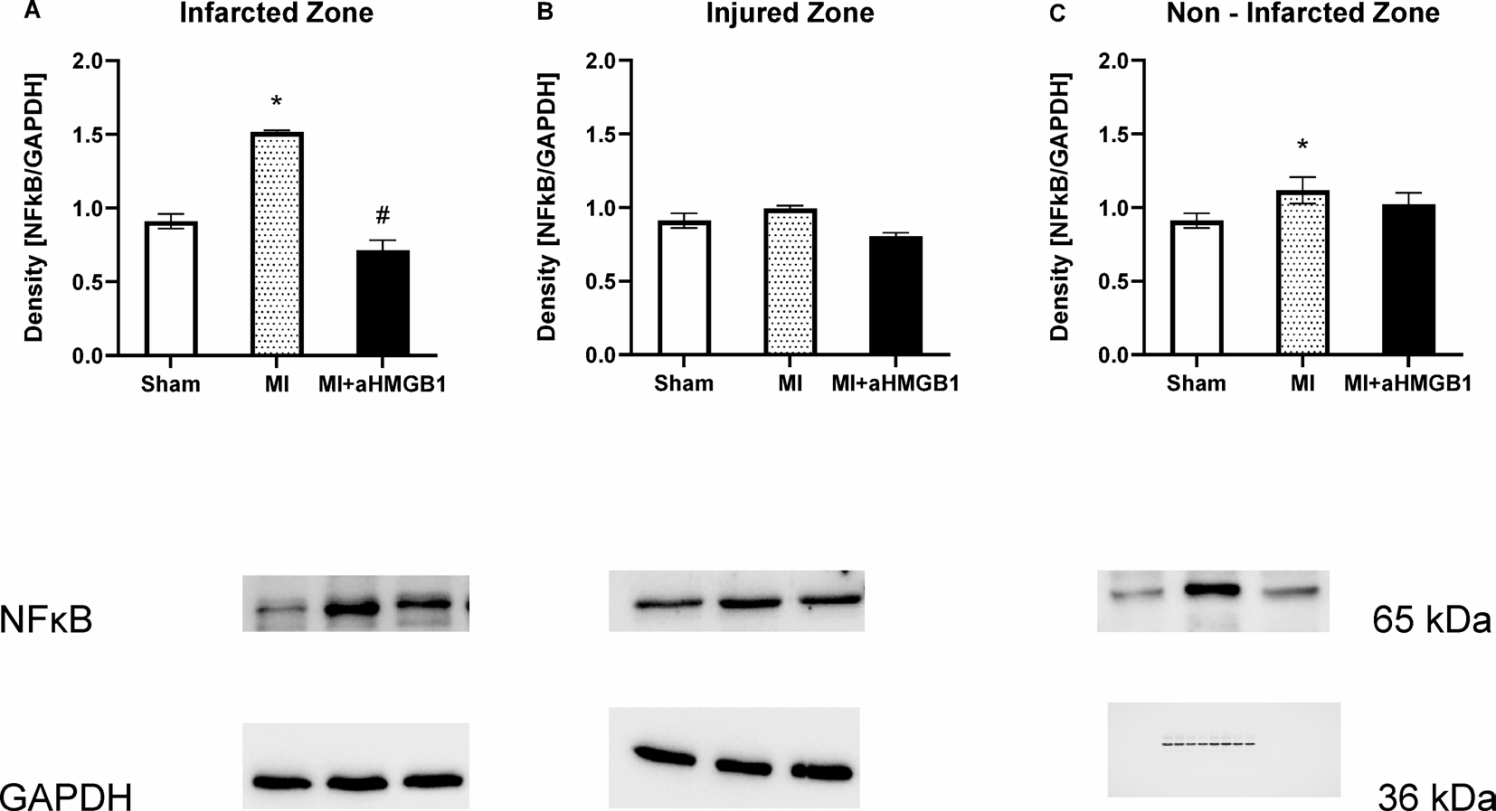
Expression of nuclear factor kappa B (NFκB) in the infarct zone (IZ; **A**), injured zone (INZ; **B**) and nonischaemic zone (NIZ; **C**) of the myocardium of sham-operated WKY rats (Sham), WKY rats with experimentally induced myocardial infarction (MI), and WKY rats with experimentally induced myocardial infarction administered anti-HMGB1 (MI + aHMGB1). Statistical significance as revealed by one-way ANOVA with subsequent Bonferroni post-hoc test when appropriate: *p < 0.01 versus Sham; #*p* < 0.01 versus MI; n = 8. The data are the means ± SEMs.

The expression of NFκB was markedly increased in the IZ and NIZ only following myocardial infarction. The administration of aHMGB1 effectively reduced this increase to levels comparable to those in the control group. No significant changes in NFκB expression were observed in other myocardial regions following either MI or aHMGB1 treatment (Fig. 5A–C).

**Fig. 6:**
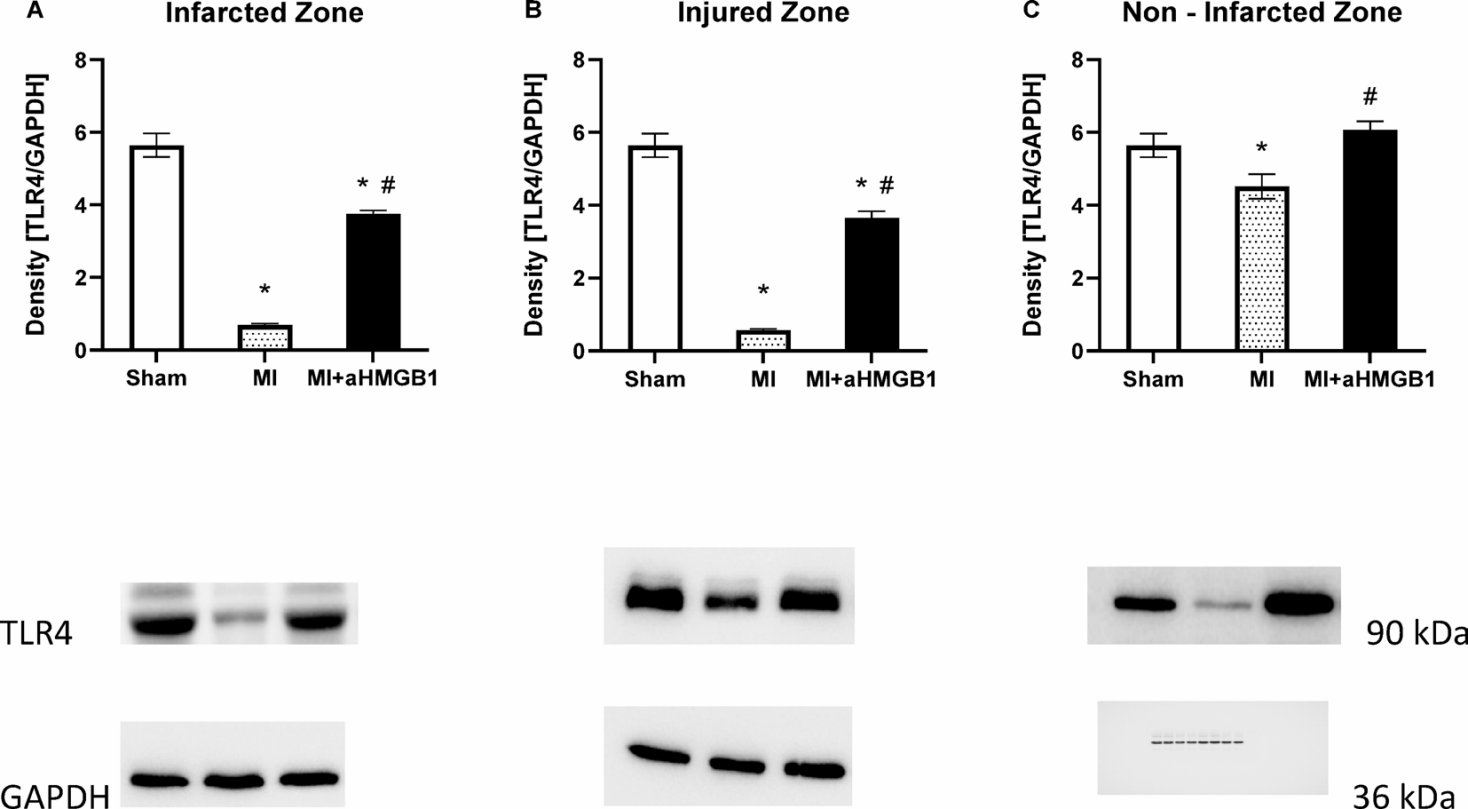
Expression of toll like receptor 4 (TLR4) in the infarct zone (IZ; **A**), injured zone (INZ; **B**) and nonischaemic zone (NIZ; **C**) of the myocardium of sham-operated WKY rats (Sham), WKY rats with experimentally induced myocardial infarction (MI), and WKY rats with experimentally induced myocardial infarction administered anti-HMGB1 (MI + aHMGB1). Statistical significance as revealed by one-way ANOVA with subsequent Bonferroni post-hoc test when appropriate: *p < 0.01 versus Sham; #*p* < 0.01 versus MI; n = 8. The data are the means ± SEMs.

The expression of TLR4 was significantly reduced in all examined myocardial regions following myocardial infarction compared to the control group. Interestingly, administration of aHMGB1 reversed this effect, resulting in a significant increase in TLR4 expression across all myocardial zones, including the infarct zone, injured zone (BZ), and nonischaemic zone (*p* < 0.01) for all comparisons (Fig. 6A–C).

### Concentrations of conjugated dienes and collagen content

**Fig. 7:**
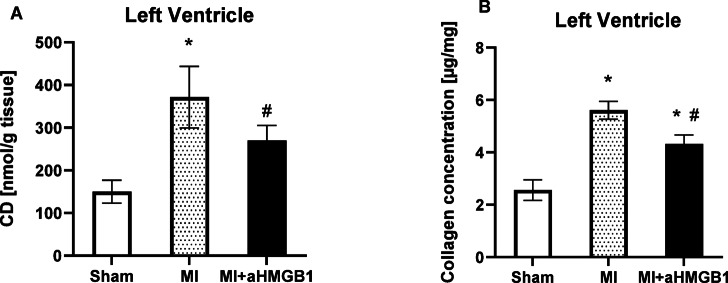
Concentration of conjugated dienes (**A**) and collagen concentration (**B**) in the left ventricles of sham-operated WKY rats (Sham), WKY rats with experimentally induced myocardial infarction (MI), and WKY rats with experimentally induced myocardial infarction administered anti-HMGB1 (MI + aHMGB1). Statistical significance as revealed by repeated measures one-way ANOVA with subsequent Bonferroni post-hoc test when appropriate: *p < 0.01 versus Sham; #*p* < 0.01 versus MI; n = 8. The data are the means ± SEMs.

A significant increase in the conjugated diene concentration was observed in the WKY + MI group compared with that in the control group, indicating enhanced lipid peroxidation following myocardial infarction. Specifically, the WKY + MI group presented a level of 371.5 ± 72 nmol/g tissue, which represents a greater than twofold increase relative to that of the control group (150 ± 27 nmol/g). This elevation reflects increased oxidative stress and membrane lipid damage in response to ischaemic injury. The administration of anti-HMGB1 led to a partial attenuation of this increase, with a concentration of conjugated dienes of 270 ± 35 nmol/g. While still elevated compared with that of the sham controls, the reduction relative to that of the untreated MI group suggests that HMGB1 contributes to the propagation of oxidative damage and that its inhibition may confer protection against postinfarction lipid peroxidation. Compared with experimental myocardial infarction, anti-HMGB1 treatment resulted in a significant reduction in the CD concentration (*p* < 0.01) (Fig. 7A). Similarly, a significant increase in myocardial collagen content was observed in the WKY + MI group compared with control group, indicating enhanced fibrotic remodeling following myocardial infarction. Particularly, the collagen concentration rose from 2.56 ± 0.39 μg/mg in the sham group to 5.61 ± 0.34 μg/mg in the WKY + MI group (*p < 0.01 vs. Sham). This increase reflects excessive collagen deposition and extracellular matrix remodeling, characteristic of post-infarction fibrosis. Administration of anti-HMGB1 protein partially mitigated this response, resulting in a value of 4.32 ± 0.34 μg/mg (*p < 0.01 vs. Sham; # *p* < 0.01 vs. MI) (Fig. 7B). Although still elevated compared with the control group, this reduction suggests that HMGB1 contributes to fibrotic signaling in the infarcted myocardium and that its neutralization can attenuate collagen accumulation. This trend mirrors the pattern observed for conjugated dienes, further supporting the link between oxidative stress and fibrotic remodeling. The concurrent attenuation of both parameters following anti-HMGB1 treatment underscores its potential in limiting post-infarction structural damage through modulation of inflammation and oxidative pathways.

## Discussion

In this study, we investigated the biochemical mechanisms and intracellular signalling pathways affected in rats during myocardial infarction, with a specific focus on how these pathways could be modulated under experimental conditions. Compared with the other studies, we did not observe any significant changes in blood pressure values across the experimental groups. Pfeffer et al.^16^ and Aires et al.^17^ reported marked reductions in BP following MI, which they attributed to compromised myocardial contractility and rhythm resulting from extensive tissue necrosis. These studies, however, constructed a model of permanent coronary ligation without reperfusion, resulting in more severe ischaemic damage. Reperfusion following ligation may limit the extent of tissue necrosis and preserve cardiac function to a greater extent. Moreover, as blood pressure was measured 7 days postsurgery, the acute effects of anaesthesia, which are known to transiently lower BP, had already subsided, contributing to the stability of the observed BP values. By this stage, compensatory neurohumoral mechanisms such as sympathetic activation, renin–angiotensin system engagement, and vascular adaptation, may have already restored systemic haemodynamic stability. This temporal factor may have contributed to the absence of detectable differences in blood pressure between groups. These differences in experimental design likely explain the discrepancy between our findings and those previously reported. Furthermore, the administration of anti-HMGB1 did not significantly alter BP values, suggesting that the HMGB1 protein does not play a major regulatory role in systemic blood pressure under these experimental conditions. The effects of HMGB1 in this context may be predominantly localised to myocardial tissue, where it influences processes such as inflammation, scar formation, and structural remodelling. Its role in directly modulating systemic vascular tone or peripheral resistance appears limited, particularly under subacute conditions. Additionally, the specific dose, timing, and route of anti-HMGB1 administration used in our study may not have been sufficient to exert a measurable influence on systemic vascular resistance or blood pressure. In a physiologically stable post-infarction animal, particularly in a reperfusion model, endogenous vascular reserves and autonomic reflexes are likely capable of buffering modest haemodynamic perturbations^18^.

Similarly, no significant differences were observed in body weight, heart weight, or kidney weight among the groups on day 7 postligation release. These findings support the notion that changes in organ structure may not manifest in the early phase post-MI when reperfusion is performed. Interestingly, we observed a trend towards decreased body weight following HMGB1 inhibition, which may reflect a subtle metabolic influence, possibly due to systemic reductions in HMGB1 levels or altered signalling pathways affecting appetite regulation^10,19,20^.

All the animals were monitored using ECG during the surgical procedure, and successful induction of MI was confirmed by ST-segment elevation. Nevertheless, to validate the occurrence of MI beyond electrocardiographic findings, we aimed to assess conventional biochemical markers of myocardial damage. Cardiac troponin T (cTnT) is widely used in both clinical and experimental cardiology applications as a specific and sensitive marker of myocardial injury^10,21^. Although anti-HMGB1 treatment produced clear anti-inflammatory and antioxidative effects in our model, aHMGB1 did not seem to affect cTnT levels, which remained elevated in both experimental groups compared with the control. We assume that anti-HMGB1, administered at the onset of reperfusion, does not prevent the immediate necrosis of already ischemically compromised cardiomyocytes but rather acts by attenuating secondary inflammatory and oxidative cascades that occur after the initial cell death.

Another aim of our study was to evaluate how MI and the subsequent administration of the anti-HMGB1 protein affects cytokine levels. We observed significant post-MI increases in the levels of the proinflammatory cytokines IL-6 and TNF-α, which aligns with findings from our previous study^22^ and other studies^23–25^. This cytokine surge is attributable not only to ischaemic insult but also to the inflammatory response triggered during reperfusion. We hypothesize that the observed increase in the serum TNF-α and IL-6 levels reflects sustained inflammation resulting from myocardial tissue necrosis and the subsequent activation of resident and infiltrating macrophages. These immune cells are known to upregulate the genes encoding these cytokines during infarct healing, leading to prolonged increases in plasma levels. Our results suggest that both IL-6 and TNF-α may serve as reliable markers of myocardial injury even 7 days postreperfusion, indicating persistent inflammatory activation. Importantly, the administration of the anti-HMGB1 antibody resulted in a marked reduction in the levels of both cytokines compared with those in untreated MI animals. These findings suggest that HMGB1 plays a pivotal upstream role in orchestrating the postinfarction inflammatory cascade. Anti-HMGB1 treatment effectively dampens the systemic inflammatory response triggered by myocardial injury. This finding agrees with studies demonstrating that HMGB1, when released extracellularly during ischaemic necrosis, acts as a potent damage-associated molecular pattern (DAMP), activating pattern recognition receptors such as TLR4 and RAGE, which in turn drive the NFκB-mediated transcription of proinflammatory cytokines^10,26^. By neutralizing HMGB1, we likely interrupt this feed‒forwards loop, leading to reduced macrophage activation and cytokine release.

The present study demonstrated that myocardial infarction induces significant alterations in NOS activity and redox homeostasis, which are partially ameliorated by the administration of the anti-HMGB1 protein. These findings support the role of HMGB1 not only as a proinflammatory DAMP but also as a modulator of endothelial function and oxidative stress during ischaemic injury. Our data revealed a marked reduction in total NOS activity in the infarct zone following MI, which is consistent with endothelial dysfunction and impaired NO bioavailability, which are hallmarks of ischaemic injury. Interestingly, no significant changes were observed in the INZ or NIZ, suggesting that the effect on NOS functionality is localized to the infarction. Anti-HMGB1 treatment significantly restored NOS activity in both the IZ and INZ, indicating its potential role vascular protection. This observation aligns with previous reports indicating that HMGB1 can suppress eNOS activity via inflammatory and oxidative pathways^25^. Moreover, anti-HMGB1 administration increased the protein expression of eNOS in cardiac and vascular tissues, further confirming its vasoprotective effects. The observed upregulation of eNOS in the MI + aHMGB1 group compared with both the MI and sham groups suggests the stimulation of HMGB1 blockade of NO synthesis capacity. Conversely, iNOS, which is typically induced under inflammatory conditions, was elevated only in the INZ following MI and was reduced by anti-HMGB1 in the INZ and IZ. These reductions suggest that while HMGB1 contributes to iNOS upregulation, other MI-associated pathways may also be involved in sustaining inflammatory NOS expression^4^. Our data revealed that NFκB expression was markedly increased in only the IZ following MI and its expression remained unchanged in the INZ and NIZ. This localized activation of NFκB may suggest a region-specific proinflammatory response in the acute phase of myocardial injury, which aligns with its well-known role as a central transcription factor in inflammation, apoptosis, and tissue remodelling. Importantly, the administration of anti-HMGB1 antibodies effectively suppressed NFκB activation in the infarcted region, reducing its expression to levels comparable to those observed in control animals. These findings support the notion that HMGB1 acts as a key upstream mediator of NFκB signalling in the ischaemic myocardium. The selective inhibition of NFκB in the IZ by aHMGB1 may reflect a reduction in DAMP-induced TLR4 mRNA signalling, which is highly active in necrotic tissue. The absence of changes in NFκB expression in noninfarcted regions further suggests that the systemic administration of aHMGB1 does not cause widespread suppression of basal inflammatory signalling, indicating a degree of regional specificity or dependence on local HMGB1 availability. These results corroborate previous studies showing that blockade of HMGB1 attenuates downstream inflammatory cascades and mitigates myocardial damage post-MI.

In this context, one of the more unexpected findings of our study was the decrease in TLR4 expression observed post-MI, followed by its apparent upregulation after anti-HMGB1 treatment. This phenomenon may be attributed to receptor trafficking and feedback regulatory mechanisms. Following ischemic injury, HMGB1 has been observed to bind to the TLR4/MD-2 complex, thereby triggering receptor internalization and transient desensitization^27^. The neutralisation of HMGB1 has been hypothesised to prevent this internalisation, leading to an increase in membrane-associated or total tissue TLR4 levels. As Hansen Selnø et al.^28^ have previously reported, a comparable compensatory upregulation of TLR4 has been observed in immune cells following HMGB1 blockade. This phenomenon may be indicative of alterations in receptor recycling, as suggested by He et al.^29^. Furthermore, in cardiac tissue, the partial TLR4 re-expression during the subacute phase of infarct healing may contribute to reparative signalling rather than proinflammatory responses^30^. It is conceivable that the presence of HMGB1 may be accompanied by an increase in the prominence of other DAMPs. These ligands have been observed to activate TLR4 independently, thus initiating autocrine or paracrine signalling loops that serve to further enhance TLR4 expression^31,32^. It is noteworthy that the internalisation of HMGB1 into endothelial cells has been demonstrated to promote angiogenic and survival pathways, at least in part through the modulation of TLR-dependent signalling. The internalisation of extracellular HMGB1 can be limited by neutralising antibodies, which may disrupt the feedback mechanisms that regulate it. This, in turn, can result in compensatory upregulation of TLR4 at the cell surface. This redistribution of receptor pools could be interpreted as a cellular attempt to restore HMGB1–TLR4 communication and maintain homeostatic signalling balance^33^.

Myocardial fibrosis, a key feature of post-infarction remodelling, is largely irreversible due to the adult heart’s limited regenerative capacity. To prevent ventricular wall rupture following myocardial infarction, rapid formation of a stable fibrotic scar is essential^34^. This process is characterized by excessive deposition of extracellular matrix components, particularly collagen. The composition and biomechanical properties of the infarct scar are critical determinants of subsequent cardiac remodeling, which can impair myocardial structure and function, ultimately contributing to heart failure^35,36^. In our study, a significant increase in myocardial collagen content was observed in the infarcted myocardium, indicating pronounced fibrotic remodeling. Treatment with anti-HMGB1 protein resulted in a partial reduction in collagen accumulation, though levels remained significantly elevated compared with sham-operated controls. This suggests that HMGB1 plays a contributory role in fibrotic signaling cascades following MI.

We suggest that aHMGB1 may promote myocardial fibrosis through activation of Toll-like receptor 4 and downstream NFκB signalling, both of which are known to induce pro-fibrotic cytokine expression and myofibroblast activation. Additionally, HMGB1 may enhance oxidative stress and facilitate the transition of fibroblasts into a profibrotic phenotype, thereby amplifying ECM production. The observed attenuation of fibrosis following HMGB1 neutralization thus supports its role as a mediator of fibrotic remodeling. These findings highlight the potential of targeting HMGB1-driven inflammatory and oxidative pathways to modulate infarct healing and improve structural outcomes following MI. Targeting the natural reparative mechanisms of myofibroblasts remains a promising therapeutic avenue for influencing scar formation and preserving myocardial function.

Although the biochemical and molecular parameters presented in our study strongly support the protective role of anti-HMGB1 treatment, we acknowledge that the absence of echocardiographic or hemodynamic measurements limits the ability to directly correlate these molecular findings with functional cardiac outcomes. Echocardiography would indeed provide essential insights into whether the observed attenuation of inflammatory and oxidative markers translates into improved systolic and diastolic function, reduced ventricular remodelling, and enhanced cardiac output. Future studies will include these functional assessments to confirm the translation of molecular changes into measurable improvements in cardiac performance.

The dual role of HMGB1 in myocardial injury appears to be closely linked to both the experimental model and the inflammatory context. In models of ischemia/reperfusion, the temporary occlusion of the coronary arteries, followed by reperfusion, has been shown to more accurately replicate clinical interventions such as angioplasty. However, the reperfusion phase introduces secondary injury mechanisms, including excessive inflammation and the generation of reactive oxygen species, which amplify HMGB1 release and promote tissue damage^9^. Conversely, permanent ligation produces a larger but more stable infarct area resulting from sustained ischemia, with a slower inflammatory progression that may favour HMGB1-mediated tissue repair and angiogenesis during later phases^10^. These discrepancies underscore the multifaceted role of HMGB1, which can function as both a pro-regenerative and pro-angiogenic agent at low concentrations, and a pro-inflammatory agent at high concentrations during the acute inflammatory phase. Furthermore, the dose dependency and the nature of the inflammatory response in each MI model have been shown to critically influence whether HMGB1 signalling drives detrimental inflammation or facilitates healing. The present findings are consistent with this context-dependent paradigm, suggesting that modulation of HMGB1 activity may shift the balance between tissue injury and repair following myocardial infarction. Our results provide further support for the dual role of HMGB1 in the pathophysiology of myocardial infarction and highlight its potential as a therapeutic target not only for reducing inflammation and oxidative stress, but also for limiting adverse fibrotic remodeling of the myocardium.

## Conclusion

This study provides evidence that myocardial infarction induces significant inflammatory and oxidative changes, particularly in the infarcted region of the myocardium. Administration of the anti-HMGB1 protein effectively attenuated these responses, as demonstrated by the reduced levels of proinflammatory cytokines (IL-6 and TNF-α), decreased NFκB activation, and modulation of NOS activity. Specifically, anti-HMGB1 treatment restored eNOS expression and activity while suppressing iNOS, suggesting improved endothelial function and reduced inflammation. The parallel reduction of both oxidative and fibrotic markers following anti-HMGB1 treatment supports a mechanistic link between oxidative stress and fibrotic remodeling, potentially mediated by HMGB1-associated inflammatory signaling. Overall, our findings suggest that HMGB1 not only amplifies oxidative damage but also contributes to pathological structural remodeling of the myocardium. Its inhibition may thus represent a promising therapeutic strategy aimed at preserving myocardial integrity by modulating both redox imbalance and fibrotic progression in the post-infarction setting.

## Limitations of the study

The limitation of this study is the lack of data regarding cardiac function. Echocardiography would have provided a more reliable assessment of cardiac function; however, at the time of the experiments, it was not possible to perform echocardiograms at the Institute, and it was not feasible to transfer the animals to another facility for this procedure owing to COVID-19 restrictions.

## Supplementary Information

Below is the link to the electronic supplementary material.

Supplementary Material 1

## Data Availability

The data that support the findings of this study are available from the corresponding author upon reasonable request. Some data may not be made available because of privacy or ethical restrictions.
