# Supplementary material for: Role of HMGB1 in ischemia/reperfusion injury in a rat model of myocardial infarction
Source: Sci Rep. 2025 Nov 25;15:44440. doi: 10.1038/s41598-025-28160-w (PMC12739116; doi:10.1038/s41598-025-28160-w)

**A full membranel scan of the blots for eNOS, iNOS, NFκB, and TLR4 in the individual myocardial zones (IZ-infarct zone, INZ- injured zone, and NIZ- non-infarct zone). The first three columns in each blot correspond to the respective experimental groups in the following order: Sham, Myocardial Infarction, and Myocardial Infarction + anti-HMGB1 treatment.**

**eNOS IZ**


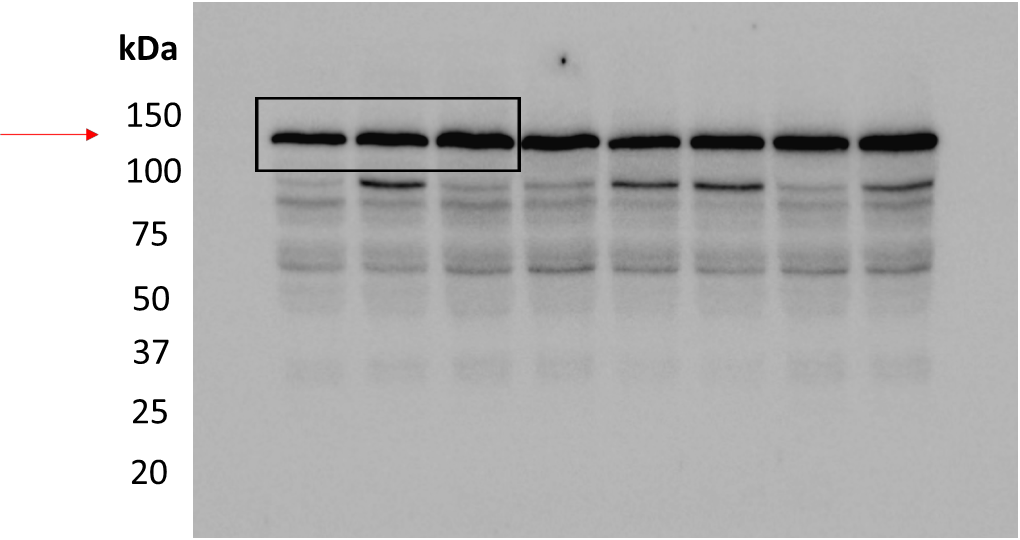


**GAPDH IZ**


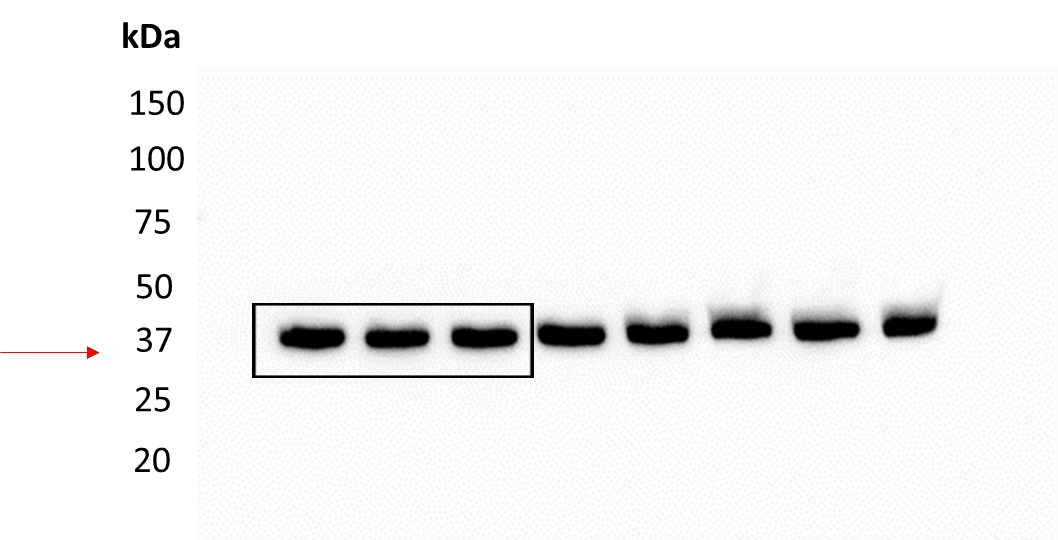


**eNOS INZ**
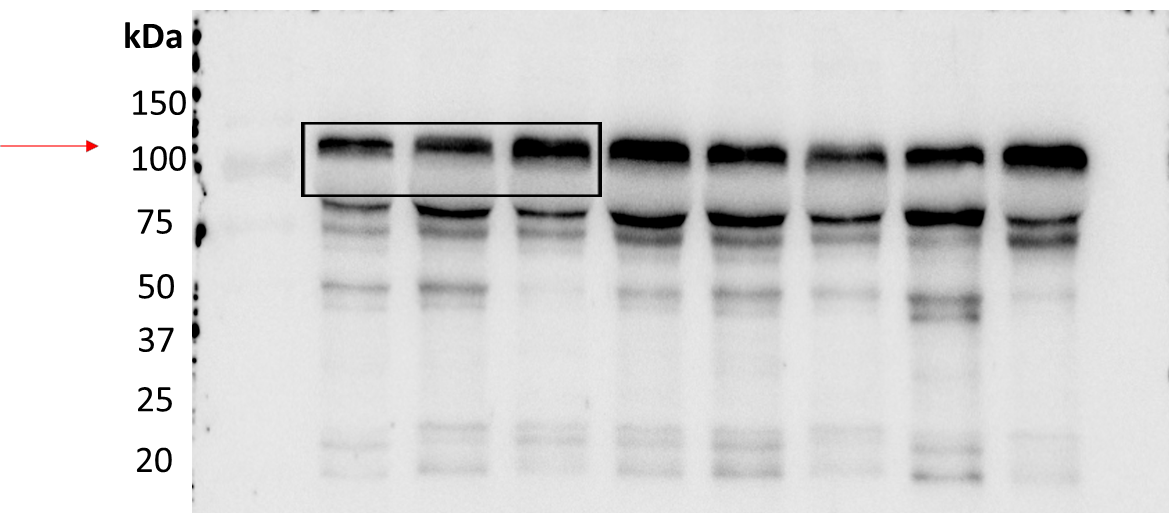


**GAPDH INZ**


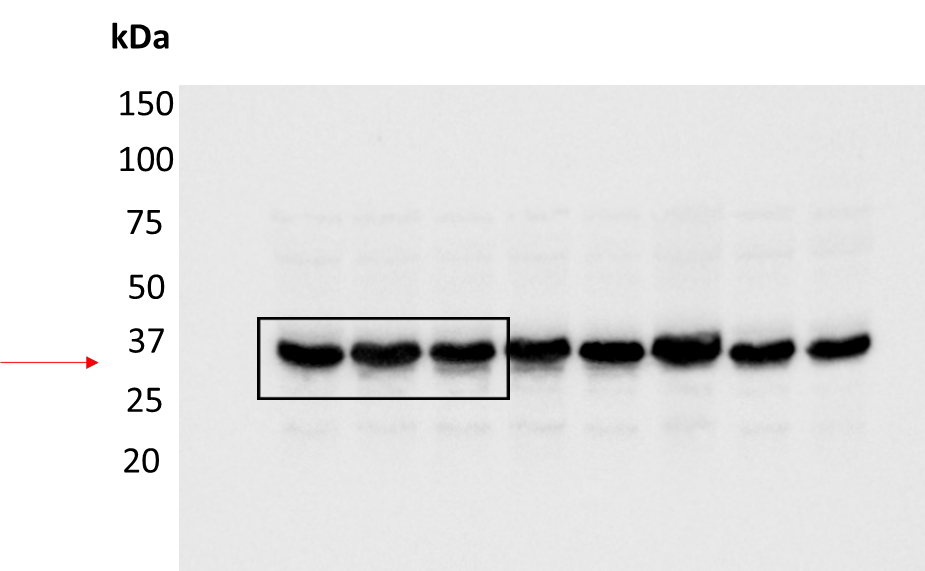


**eNOS NIZ**


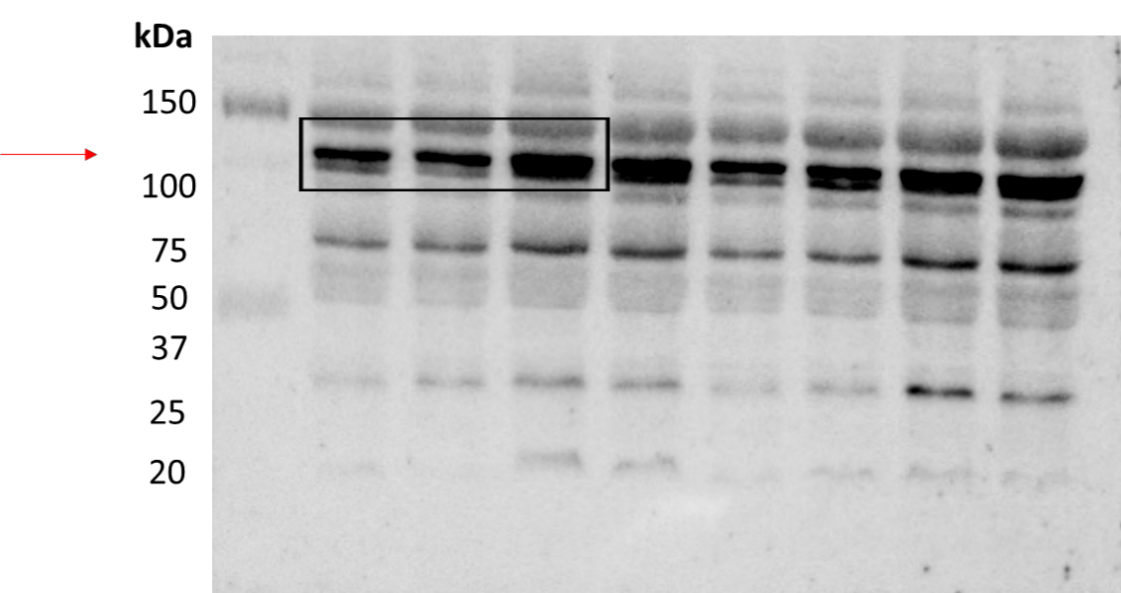


**GAPDH NIZ**


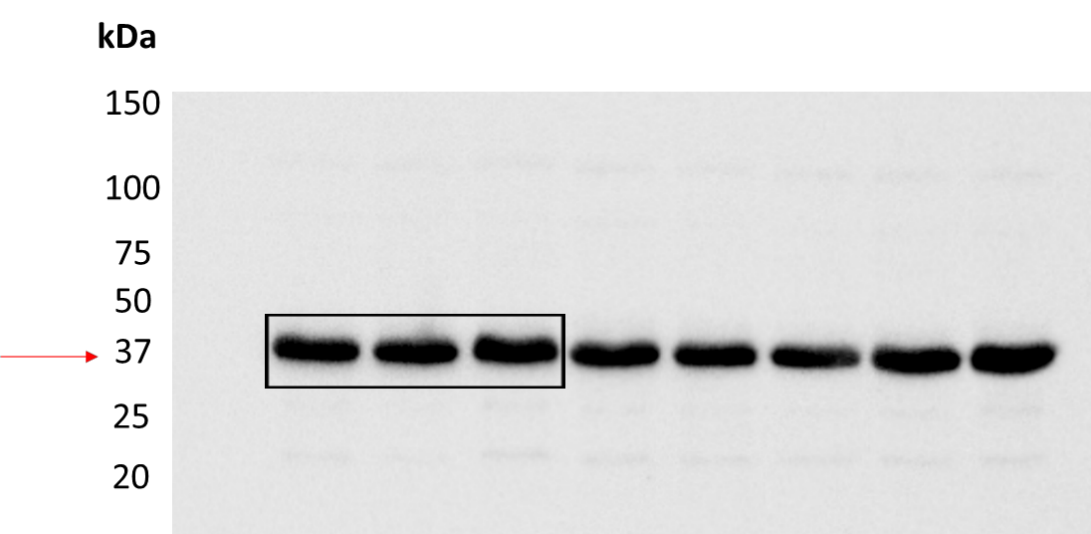


**iNOS IZ**


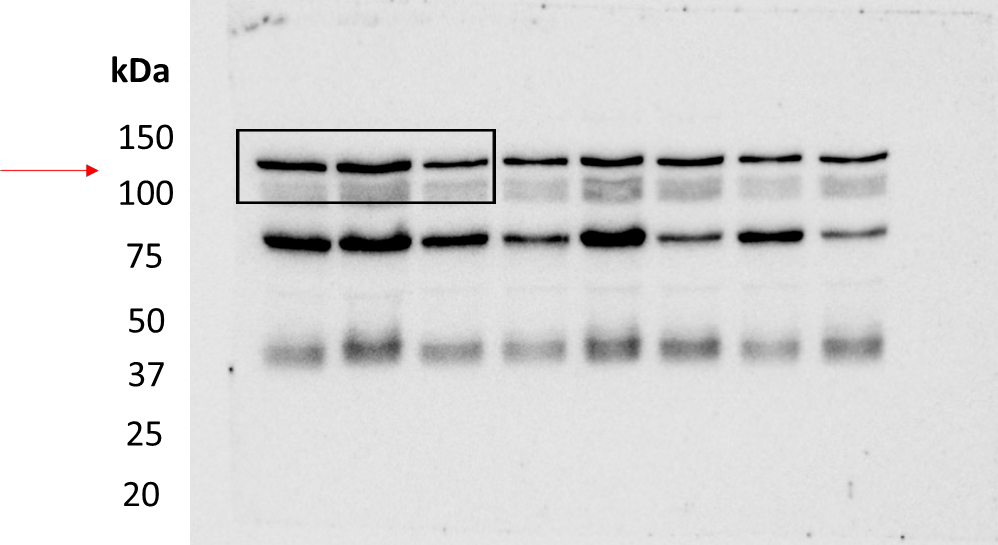


**GAPDH IZ**


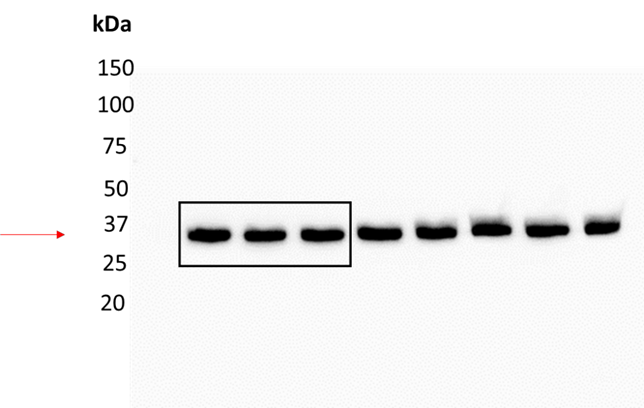


**iNOS INZ**


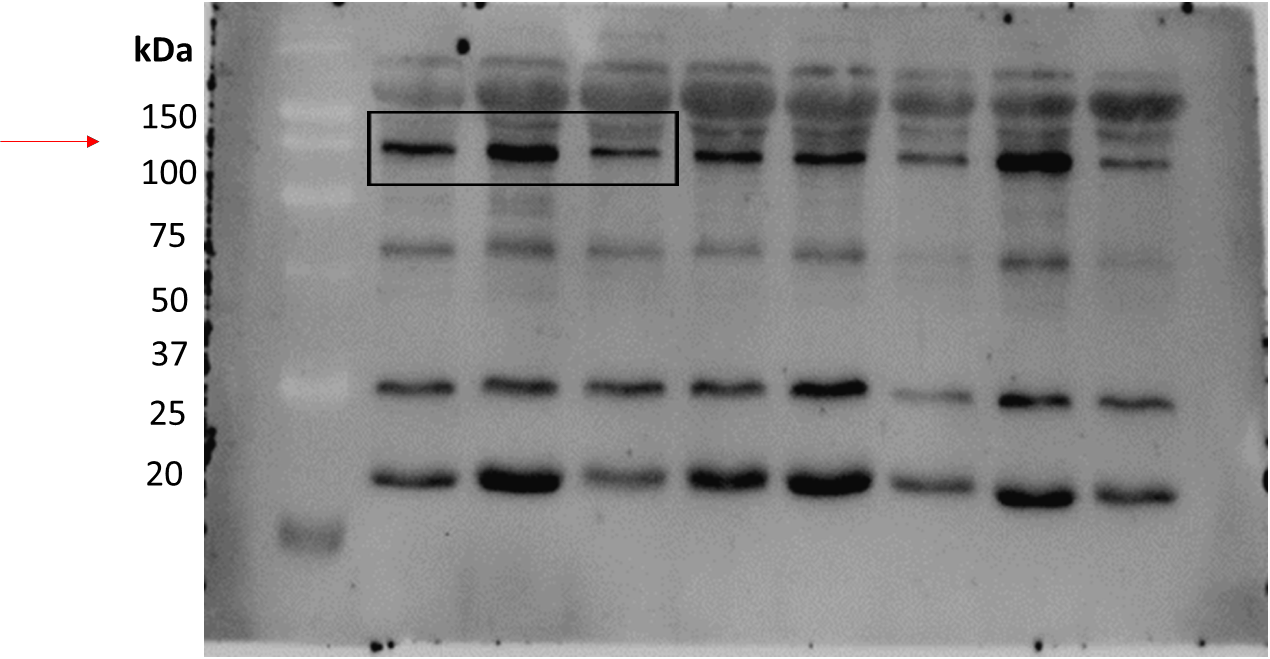


**GAPDH INZ**


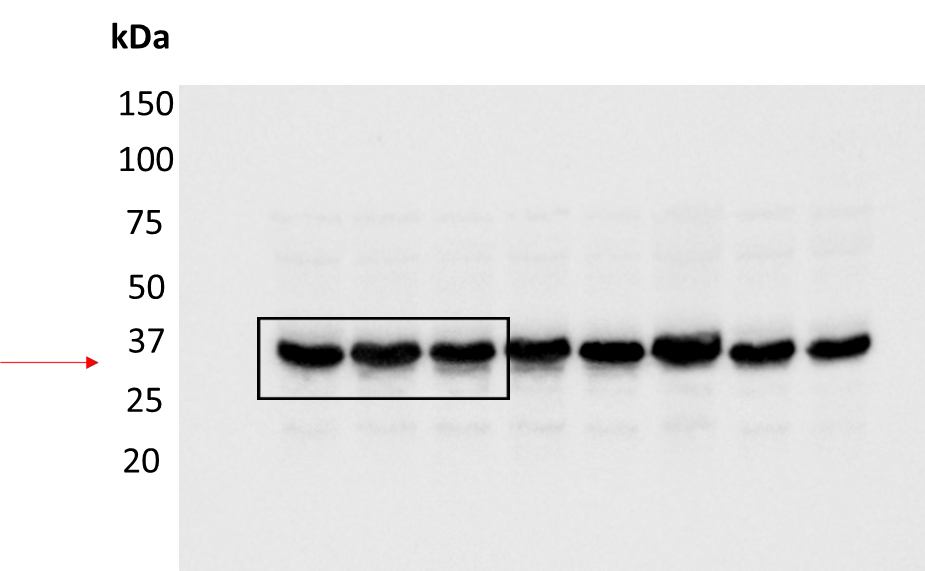


**iNOS NIZ**


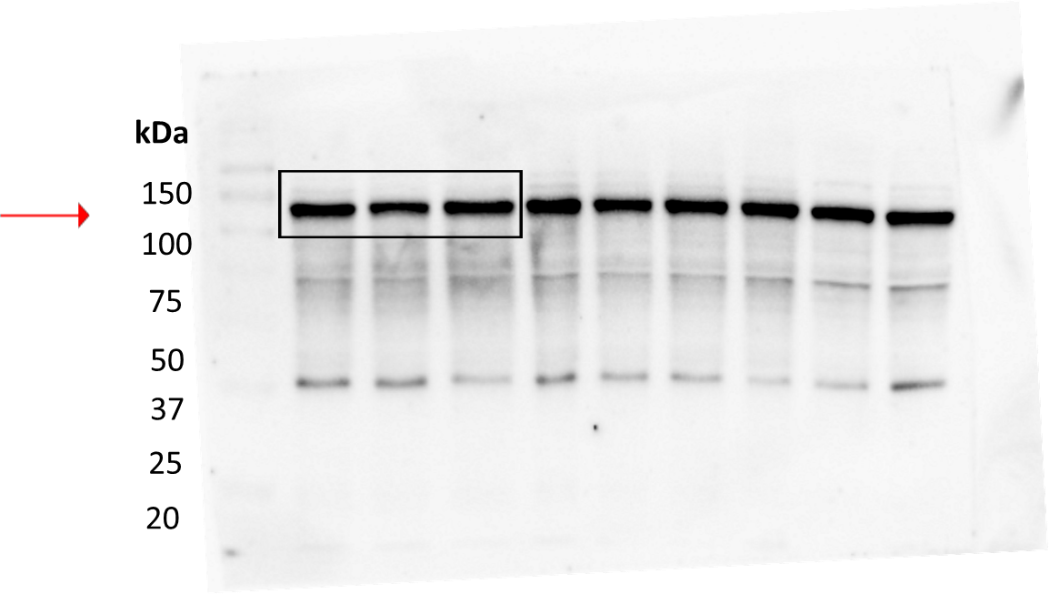


**GAPDH NIZ**


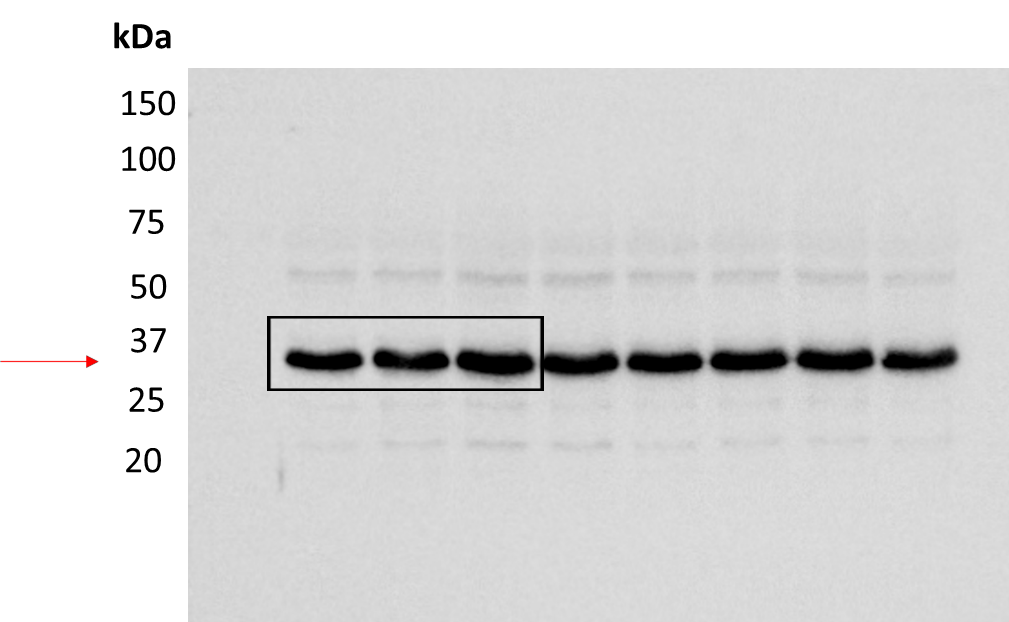


**NFκB IZ**


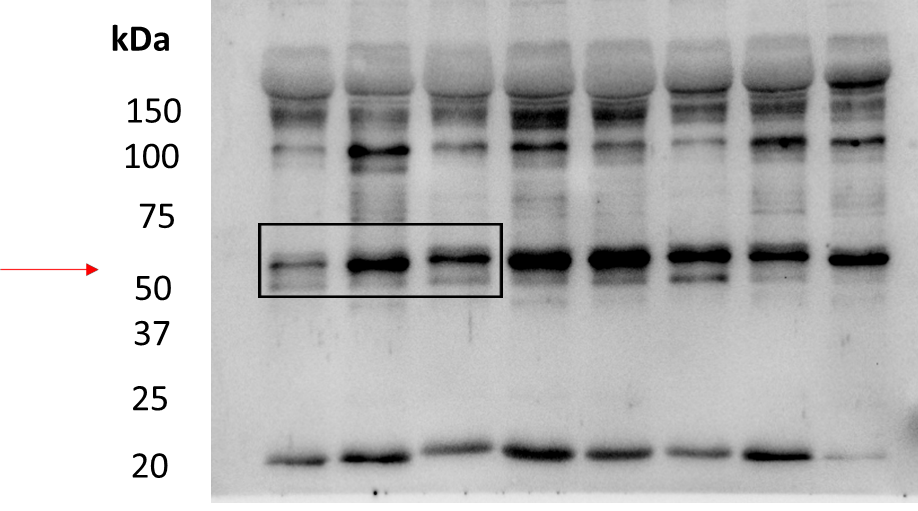


**GAPDH IZ**


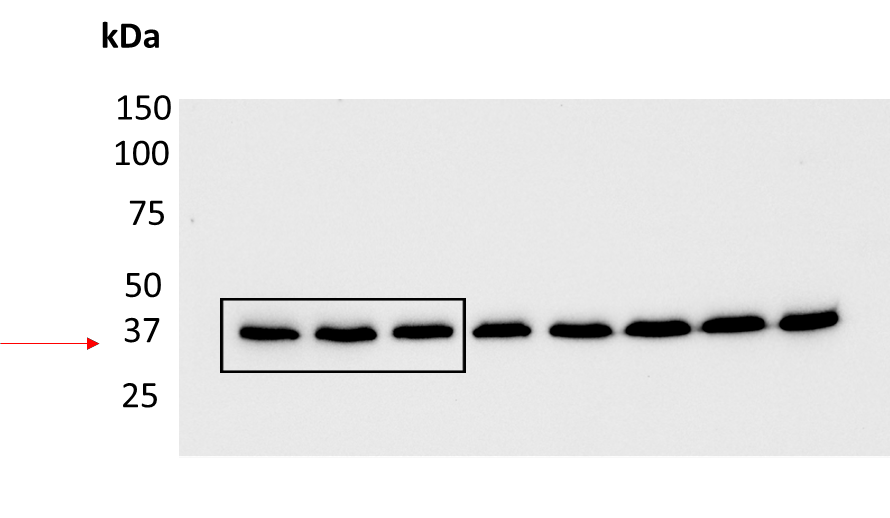


**NFκB INZ**


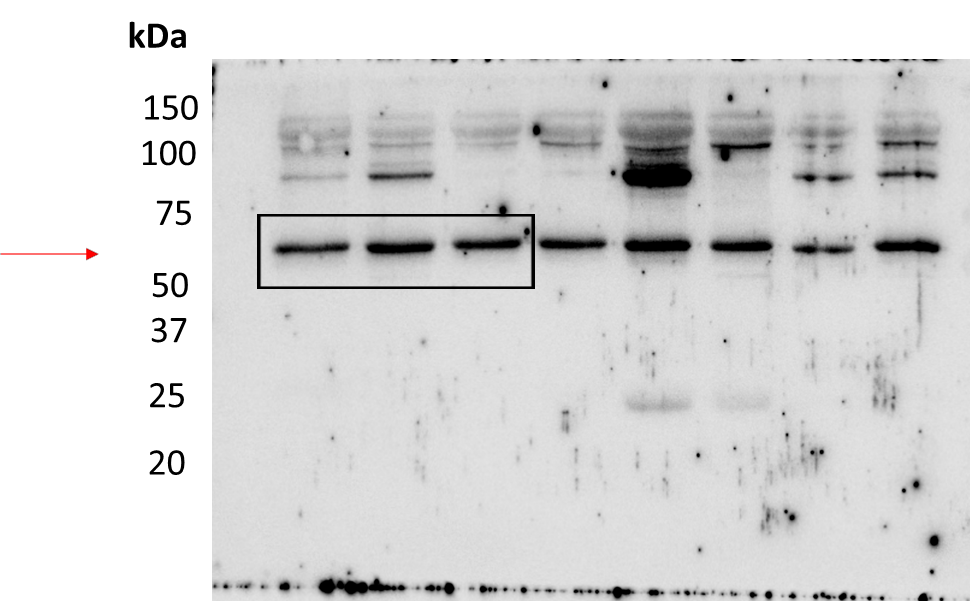


**GAPDH INZ**


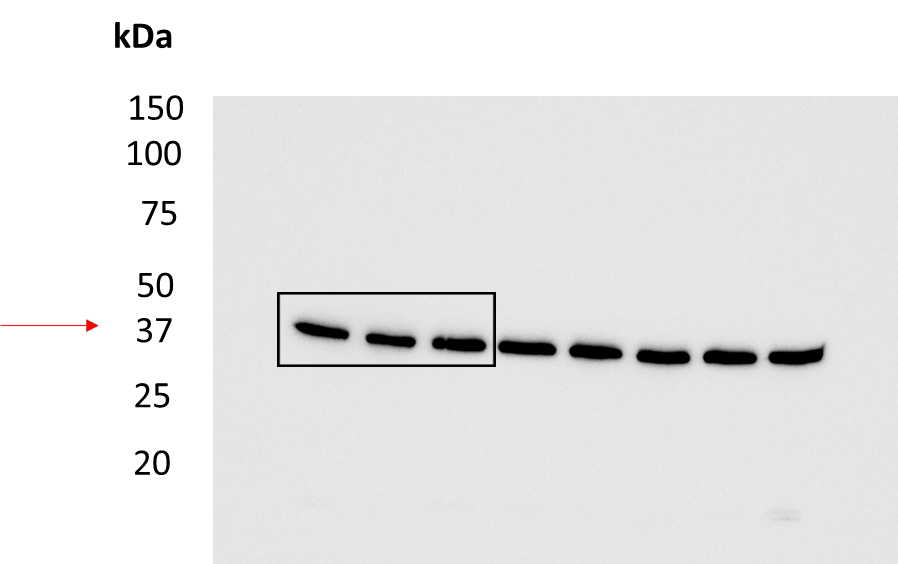


NFκB NIZ


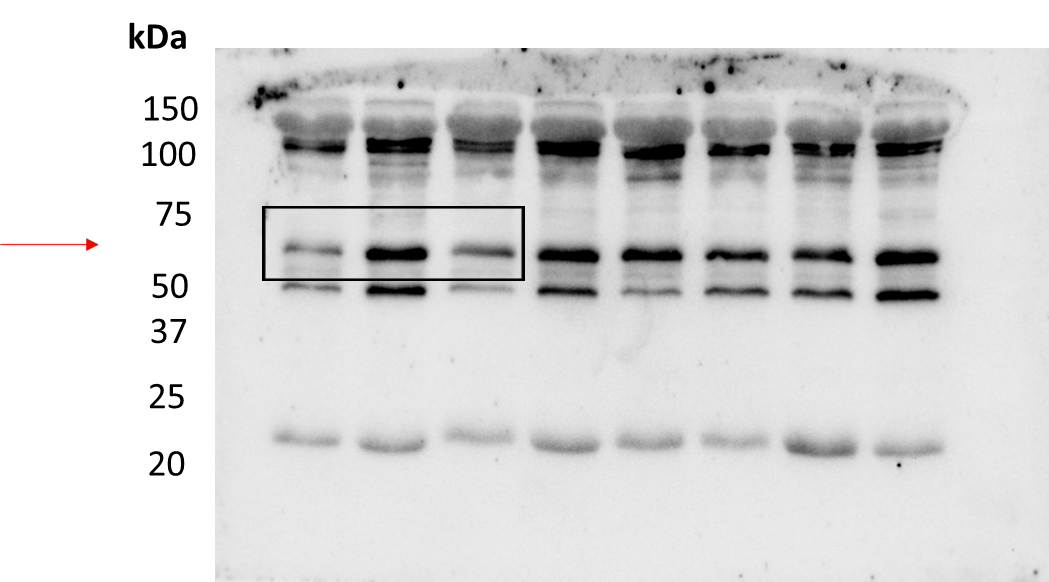


**GAPDH NIZ**


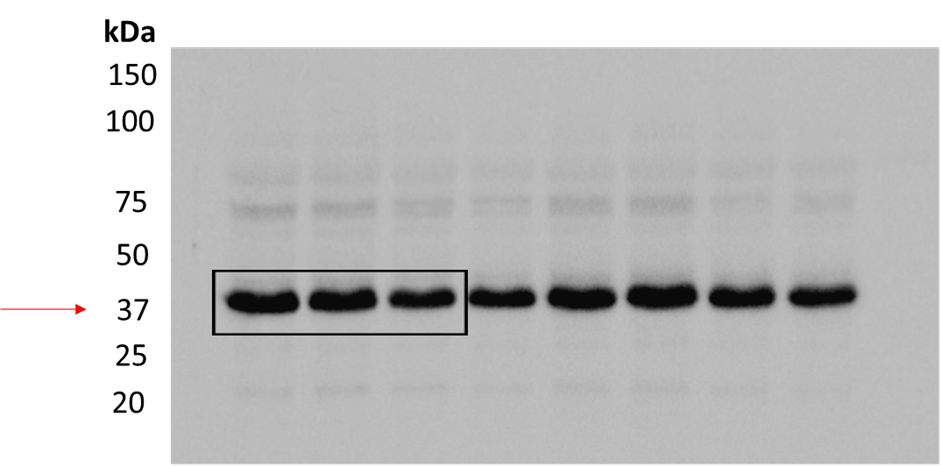


**TLR4 IZ**


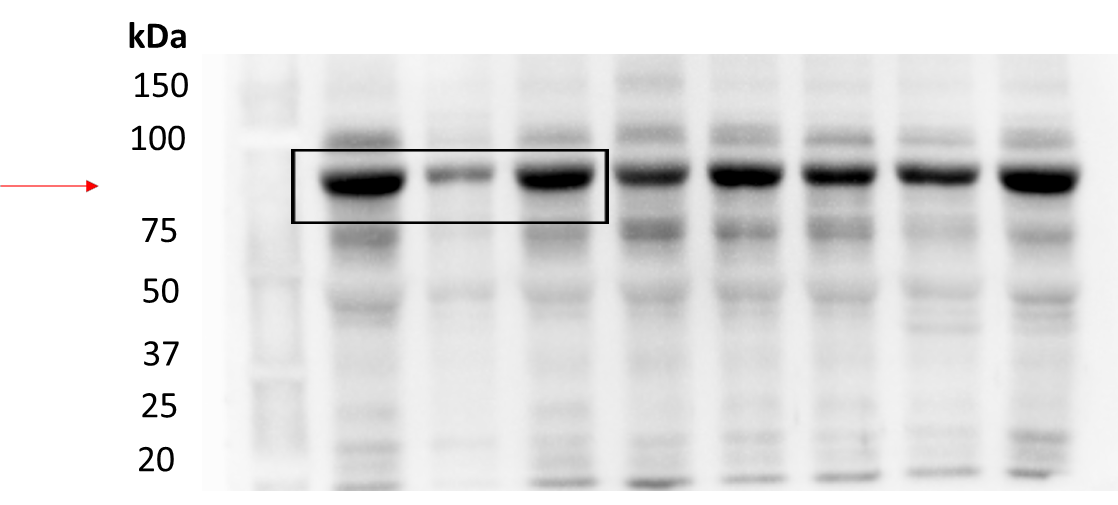


**GAPDH IZ**


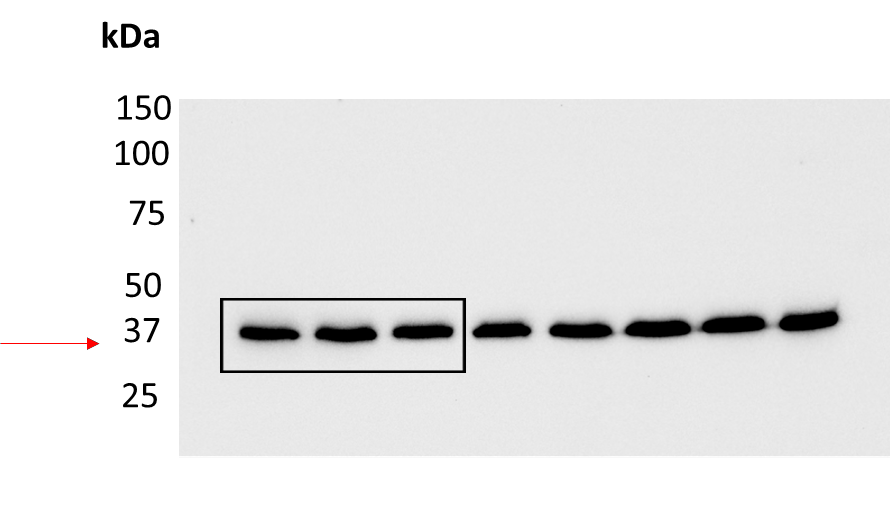


**TLR4 INZ**


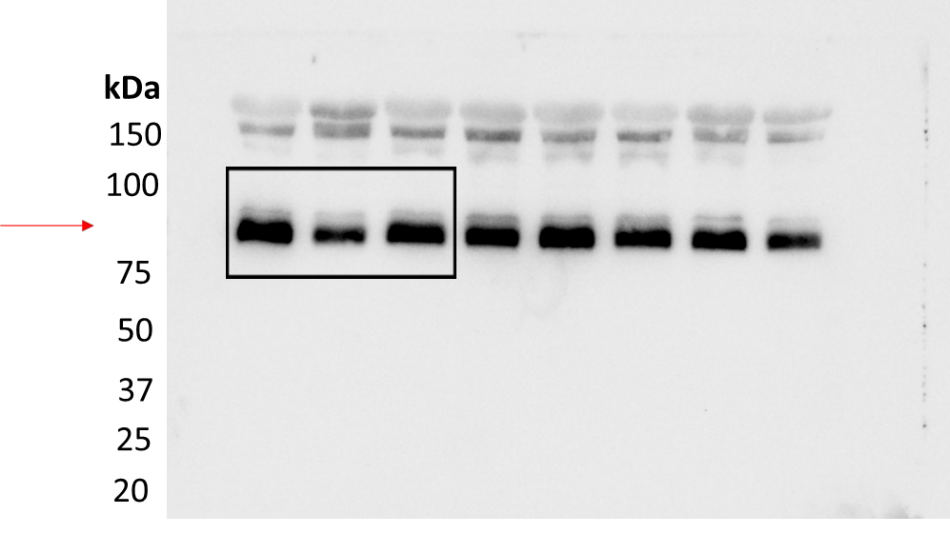


**GAPDH INZ**


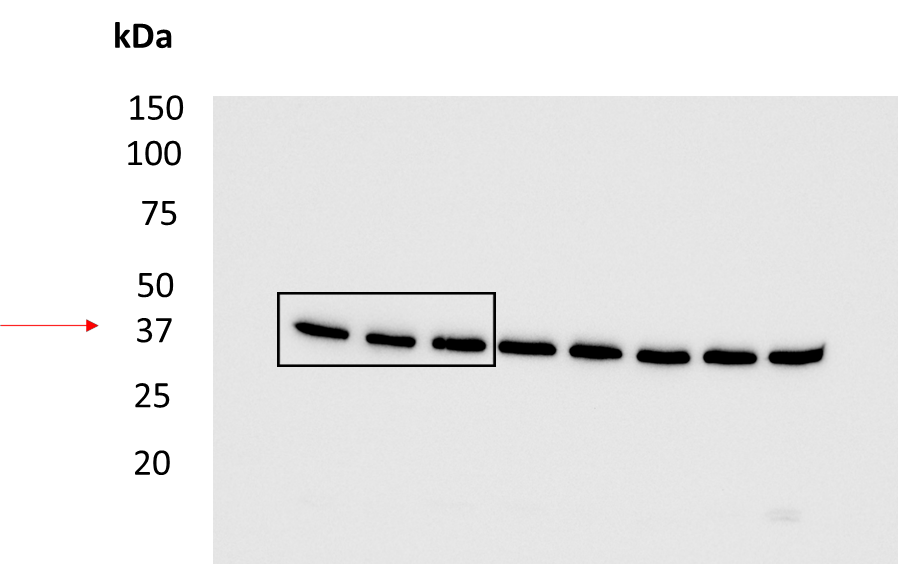


**TLR4 NIZ**


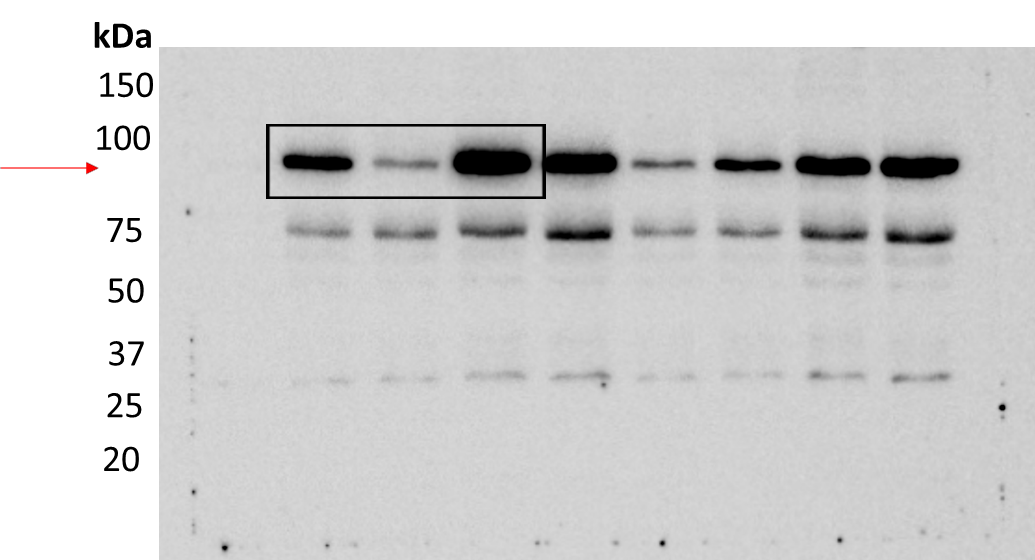


**GAPDH NIZ**


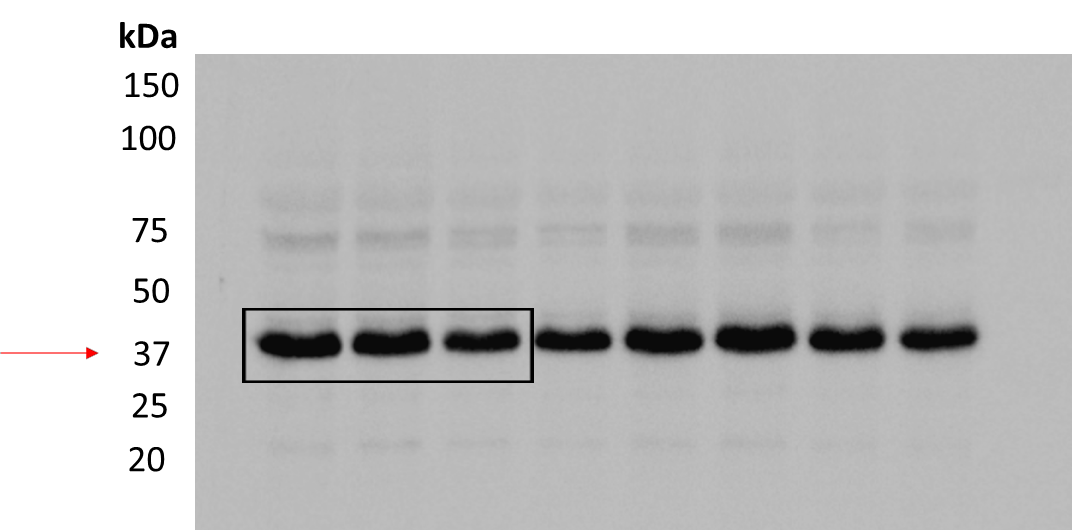

Supplement: Supplementary file 1 — Supplementary Material 1 [file 41598_2025_28160_MOESM1_ESM.docx]
